# Evaluation of Muscle Long Non-Coding RNA Profile during Rearing and Finishing Phase of Bulls Subjected to Different Prenatal Nutritional Strategies

**DOI:** 10.3390/ani14040652

**Published:** 2024-02-18

**Authors:** Roberta Cavalcante Cracco, Pamela Almeida Alexandre, Guilherme Henrique Gebim Polizel, Arícia Christofaro Fernandes, Miguel Henrique de Almeida Santana

**Affiliations:** 1 Department of Animal Science, College of Animal Science and Food Engineering—USP, Av. Duque de Caxias Norte, 225, Pirassununga 13635-900, SP, Brazil; roberta.cracco@usp.br (R.C.C.); guilhermepolizel875@gmail.com (G.H.G.P.);; 2 Microbiomes for One Systems Health (MOSH), CSIRO Agriculture & Food, 306 Carmody Rd, St Lucia, QLD 4067, Australia; pamela.alexandre@csiro.au

**Keywords:** cattle, epigenetics, fetal programming, gene expression, muscle

## Abstract

**Simple Summary:**

The study examines how different prenatal nutrition plans affect muscle development and epigenetic mechanisms in Nellore cows’ offspring. It looks at 63 male calves from cows given no supplementation (NP), partial supplementation (PP), or full supplementation (CP) during pregnancy. RNA sequencing showed no difference in epigenetic mechanisms, but did reveal 1823 transcripts at 15 months and 1533 at 22 months. Among these, a few showed differences between groups. Interestingly, while maternal nutrition didn’t affect epigenetic mechanisms directly, it seemed to influence how certain RNA molecules regulated them.

**Abstract:**

Maternal nutrition has the ability of influence critical processes in fetal life, including muscle development. Also, in this period, epigenetic sensitivity to external stimuli is higher and produces long-lasting effects. Thus, the aim of this study was to investigate epigenetic mechanisms, including the identification and characterization of long non-coding RNA (lncRNA) from animals that had undergone different strategies of prenatal supplementation. A group of Nellore cows (n = 126) were separated into three nutritional plans: NP (control)—Not Programmed, without protein–energy supplementation; PP—Partially Programmed, protein–energy supplementation in the final third of pregnancy; and CP—Complete Programming, protein–energy supplementation during the full period of gestation. A total of 63 male offspring were used in this study, of which 15 (5 per treatment) had *Longissimus thoracis* muscle at 15 (biopsy) and 22 months (slaughter). Biopsy samples were subjected to RNA extraction and sequencing. Differential expression (DE) of remodeling factors and chromatin-modifying enzyme genes were performed. For the identification and characterization of lncRNA, a series of size filters and protein coding potential tests were performed. The lncRNAs identified had their differential expression and regulatory potential tested. Regarding DE of epigenetic mechanisms, no differentially expressed gene was found (*p* > 0.1). Identification of potential lncRNA was successful, identifying 1823 transcripts at 15 months and 1533 at 22 months. Among these, four were considered differentially expressed between treatments at 15 months and 6 were differentially expressed at 22 months. Yet, when testing regulatory potential, 13 lncRNAs were considered key regulators in the PP group, and 17 in the CP group. PP group lncRNAs possibly regulate fat-cell differentiation, in utero embryonic development, and transforming growth factor beta receptor, whereas lncRNA in the CP group regulates in utero embryonic development, fat-cell differentiation and vasculogenesis. Maternal nutrition had no effect on differential expression of epigenetic mechanisms; however, it seems to impair lncRNA regulation of epigenetics.

## 1. Introduction

The main product in beef-cattle production is meat. In this setting, muscle development is highlighted, and it is of global interest to find out more about the mechanisms that act on it and that can be manipulated in order to produce meat in a more efficient way. However, poor maternal nutrition is a common scenario in beef-cattle production, in which the cow is usually managed under an extensive production system, depending only on pastures for feed availability [1]. In this case, maternal dietary intake can influence processes critical in fetal and embryonic development, even though the nutrient requirement for the conceptus is negligible in the earliest stages of gestation [2]. These processes can predispose offspring to altered endocrine regulation of growth and maintenance, which is associated with other metabolic dysregulations later in life, as a long-term consequence of fetal programming [3,4]. Throughout fetal development, the conceptus relies on maternal nutrients for sustenance. Nevertheless, the development of essential organs like the brain and heart have priority, leaving fetal skeletal muscle growth contingent upon nutrient availability [5,6,7]. The intrauterine phase is particularly critical for skeletal muscle development, as there is no net increase in muscle fiber count after birth [8,9,10]. The phenotypic and molecular influence of nutrition on dams and their offspring is closely related to epigenetics.

Epigenetics is defined as the set of heritable changes in gene expression, without any change in the genetic code, which can be altered by environmental factors, and are the primary mechanisms through which the effects of fetal programming are carried out [11]. There is growing evidence that nutritional conditions can alter genome activity through epigenetic modifications [12,13,14]. Although epigenetic sensitivity persists throughout life, there are periods when it is higher and produces longer-lasting effects. [15]. Many of these critical periods, particularly in mammals, overlap with the periods when resource transfer between mother and progeny occurs, either through the placenta or breast milk [16]. Epigenetic modifications include DNA methylation, histone modifications, and non-coding RNA such as microRNA [17,18] and long non-coding RNA (lncRNA).

The lncRNA molecules are characterized by having a size greater than 200 nucleotides, having a very low coding potential, being poorly conserved between species, and also not having a specific pattern in their sequence, which makes them difficult to categorize and increases the difficulty of predicting their function. [19]. The majority of lncRNA that has already been characterized is generated by the same transcriptional machinery as other messenger RNAs (mRNA; [20]). Also, these transcripts have a 5′ terminal methylguanosine cap and are polyadenylated. The regulatory role of lncRNA in epigenetics is linked to chromatin-modifying proteins, and it recruits them to specific sites in the genome to modulate chromatin state and impair gene expressions [21].

Finally, there are reports in the literature that maternal nutrition can impact fetal development, even without notable phenotypic differences [22]. Thus, the hypothesis of this work is that there are epigenetic mechanisms acting silently in the muscular development of certain cattle that underwent different nutritional strategies during their fetal life. Thus, the objectives of this work were (1) to test the differential expression of genes related to epigenetic mechanisms and (2) to identify and characterize lncRNA using RNA-Seq data from animals that underwent different strategies of prenatal supplementation.

## 2. Material and Methods

### 2.1. Ethics Statement

This study was approved by the Research Ethics Committee of FZEA/USP on March 10, 2018, under protocol No. 1843241117, and according to the guidelines of the National Council for the Control of Animal Experimentation (CONCEA).

### 2.2. Experimental Design

A group of 126 Nellore dams were fixed-time artificially inseminated (FTAI) with the semen of four bulls with known genetic value. Pregnancy diagnosis was taken at 30 days after FTAI, and the animals were then separated into three treatments: NP—Not Programmed, without protein–energy supplementation (control); PP—Partially Programmed, protein–energy supplementation in the final third of pregnancy; and CP—Complete Programming, protein–energy supplementation during the complete period of gestation. All groups received a 0.03% live-weight mineral supplementation; PP and CP animals received protein–energy supplementation at the level of 0.3% live weight (composition and nutrients are shown in Table 1) [23], and a mineral supplement was already included in it (more details about the dams’ phenotypes during the pregnancy period can be found in [23]. Briefly, dams were blocked into the groups based on age, body weight, and body-condition score. Animals were allocated to pasture paddocks of *Urochloa brizantha cv.* Marandu, with access to the supplement and to water ad libitum. In contrast with the initial period (no phenotype differences among the treatments), the cows showed phenotype differences in the pre-delivery period (i.e., body weight, body condition score and rump fat thickness) among the treatments. This was associated with the fact that there had been different prenatal nutrition strategies employed.

After calving, all animals remained together until weaning (average of 220 days old), regardless of the treatment, and protein–energy supplementation ceased. The animals were subjected to the same sanitary, vaccination, and feeding protocols already implemented on the farm where the experiment was conducted. After weaning, the animals were divided by sex, regardless of treatment, and placed in separate pastures, where they remained throughout the rearing phase. More details about the rearing phase, including management, evaluations, and sample collection, can be found in [24]. Young bulls remained on the pasture until the beginning of the finishing phase, at 19 months.

**Figure 1:**
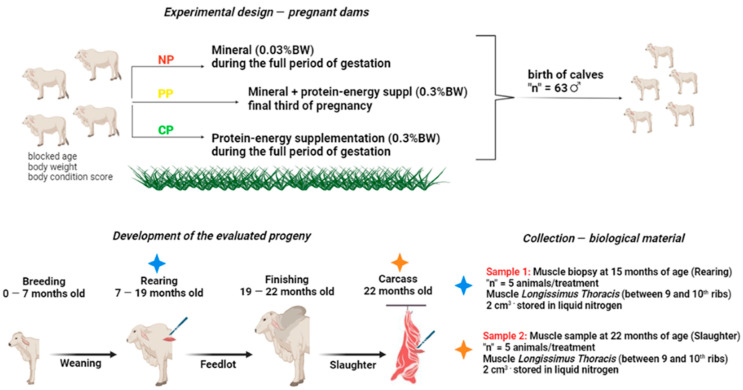
Schematic of the experimental design and the biological samples collected in the experiment.

Young bulls were finished in a feedlot system for 106 days (15 days of adaptation period, and 91 days of effective feedlot). During this period, they received three different diets: an adaptation diet was provided in the first 15 days; a second diet for 35 days; and a third diet for 56 days (Figure 1). At the end of the finishing phase, animals were slaughtered at the FZEA/USP school slaughterhouse, located approximately 500 m from the feedlot installations. More details about the finishing phase and slaughter can be found in [25].

### 2.3. Sample Collection and RNA Extraction and Sequencing

At slaughter (676 ± 28 days of age), samples of approximately 2 cm^3^ were collected from the *Longissimus* muscle (between 9 and 10th ribs), cut into smaller pieces using a scalpel, and rapidly stored in liquid nitrogen until the moment of RNA extraction. Samples of 5 progenies from the same sire were randomly selected within each treatment for sequencing at both 15 and 22 months of age (totaling 30 samples). About 80 milligrams of each sample was macerated in nitrogen with a crucible and pestle, and extraction was performed using TRIzol (Invitrogen, Carlsbad, CA, USA), following the manufacturer’s protocol. The concentration and quality obtained at the end of the extraction were evaluated using a spectrophotometer (NanoDrop 2000, ThermoScientific, Waltham, MA, USA), analyzing the ratios A260/280 and A260/230. The samples that showed undesirable parameters were re-extracted.

RNA integrity (RIN) was obtained using the Bioanalyzer 2100 equipment with Labchips RNA 6000 Nano, following the manufacturer’s guidelines (Agilent Technologies Ireland, Dublin, Ireland), and all samples had an RIN value greater than 7.0. For the construction of the RNA libraries, the TruSeq™ RNA Sample Prep kit (Illumina, San Diego, CA, USA, 2012, Part # 15026495 Rev. D) was used according to the instructions associated with TruSeq^®^ RNA Sample Preparation v2. The libraries were sequenced on the Illumina HiSeq 2500 instrument using the TruSeq PE Cluster Kit and the TruSeq SBS Kit (2 × 100 bp).

**Figure 2:**
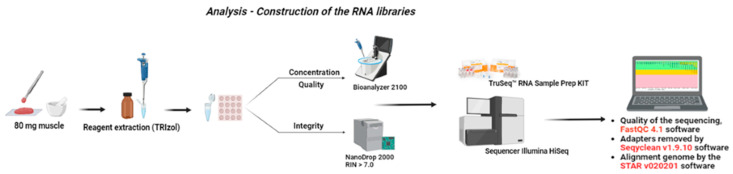
Diagram of RNA data processing, sequencing, and filtering.

To determine the quality of the sequencing, FastQC 4.1 software (http://www.bioinformatics.babraham.ac.uk/projects/fastqc/, accessed on 5 July 2023) was used. Then, adapters inserted during library formation were removed by Seqyclean v1.9.10 software [26]. The alignment of the samples to the *Bos taurus* reference genome (ARS-UCD1.2.95) was performed by the STAR v020201 software [27], with default parameters (Figure 2) and using an annotation file (ARS-UCD1.2.95), and this generated a file with the number of reads paired to each gene (counts).

### 2.4. Expression of Genes Related to Epigenetic Mechanisms

We selected 164 genes related to remodeling factors and chromatin-modifying enzymes (Supplementary Materials). After aligning the samples to the genome and obtaining the read counts for the genes of interest, we performed the analyses of differentially expressed epigenetic genes by contrasting each group to the others. EdgeR v3.32.0 [28] and Limma v3.46.0 [29] packages were used, both in the R statistical environment. The read-counts file of the genes of interest and a file containing factors for normalization (sample number, treatment, age of dam, and age of animal) were used to assemble the comparison matrix. Afterward, the steps presented by [30] were followed.

### 2.5. lncRNA Differential Expression

After aligning the reads of each sample to the reference genome (ARS-UCD1.2), the Cufflinks software v. 2.2.1 [31] was used to generate an annotation file for each sample, using the reference annotation. Individual annotation files and the bovine reference annotation were then merged into one file using Cuffmerge. Through the genomic position of the transcripts, it was possible to select those having the potential to be lncRNA. Only transcripts from class codes “i” (intron transcripts, “j” (new isoforms), “o” (generic overlap with known exon), “u” (intergenic transcripts), and “x” (overlap with known gene on the opposite strand) were selected. From there, a FASTA file was generated containing the sequence of transcripts that had passed through size filters (>200 base pairs [bp]) and an open reading frame size (ORF; <300 bp), using GetOrf software v. 1.0. Absence of protein homology was determined through testing with BLASTx [32] and coding potential was determined using CPC2 [33]. Transcripts that passed through the filters were considered new lncRNAs. In order to generate the read-counts table for the new lncRNA, FeatureCounts [34] was employed. The edgeR package was used in the R environment in order to test the differential expression between the three treatments in the identified lncRNAs; those with a Q value < 0.05 were considered differentially expressed. To characterize the differentially expressed lncRNAs, a search for homology was performed using BLAST+ [35] in the NONCODE database [36]; homologies with an E value > 10^−6^ were considered significant, as described by [37].

### 2.6. Regulatory Potential and Co-Expression Networks

To identify regulatory genes related to fetal programming and generate a co-expression network, from 14,125 genes expressed in muscle across all the samples, 1222 were selected for having Gene Ontology [38] terms associated with skeletal muscle (GO:0048641, GO:0048630, GO:0048631, GO:0048741, GO:0003009, GO:0003010, GO:0003011, GO:0043501, GO:0043503, GO:0043403, GO:0007519, GO:0035914, GO:0014856, GO:0014734, GO:0014732, GO:1904204, GO:0014816, GO:0048644, and GO:0048634). These genes were considered targets in a regulatory impact factor (RIF) [39] analysis which tested the potential of the lncRNA to be a key regulator of epigenetic modeling, contrasting the treatments with the control. This algorithm assumes that master regulators in a network contribute to the alteration of gene expression by changing their behavior in different biological conditions. To try to predict the role of lncRNA in muscle development in animals that had undergone fetal programming, co-expression networks were constructed for each treatment using 1222 mRNA and 394 lncRNA, using the partial correlation and information theory algorithm (PCIT package from the R statistical environment; [40,41]). After the execution of the PCIT, the filtering between the groups was performed according to the following table (Table 2). The Cytoscape software v 3.10.1 [42] was used to build the co-expression networks, and DAVID [43] was used for functional enrichment.

## 3. Results

### 3.1. Differential Expression of Epigenetic Mechanism’s Genes

No gene related to the epigenetic mechanism was differentially expressed between treatments at any time. All had a *p*-value of > 0.1.

### 3.2. Identification of New lncRNA

After selecting transcripts through their class code, 68,316 new transcripts were identified at 15 months of age and 62,573 new transcripts were identified at 22 months of age, all with the potential to be new lncRNA. Of these, 88.1% and 89.7% of transcripts (15 and 22 months of age, respectively) belonged to class code “j”, followed by 7.6% and 6.4% of transcripts (in their respective ages, as above) belonging to the class code “u”.

When applying the sequential filters, 99.9% of the transcripts in both ages were larger than 200 nucleotides. The next filter, which required transcripts to have an ORF smaller than 300 bp, was the one that excluded the most, leaving only 7.4% and 7.0% (15 m and 22 m, respectively) of the initial transcripts. After this step, 1.7% of the initial transcripts were excluded at both ages because of similarities to the UniProt database, and 15 transcripts at 15 months and 19 at 22 months were excluded because of their coding potential according to CPC2. Finally, an exon filter was applied, excluding 3.0% and 2.8% of the initial transcripts, and leaving only 1823 transcripts (2.7% of the initial amount) at 15 months of age and 1533 transcripts (2.5%) at 22 months.

### 3.3. Differentially Expressed lncRNA

When looking at the adjusted *p*-values, only one transcript was considered differentially expressed between the groups at both times (TCONS_00092235, at 22 months for NP vs. CP contrast). However, given the exploratory nature of the study, transcripts with *p*-values < 0.01 were also considered; these totaled ten transcripts, four of which appeared at 15 months, and there were six transcripts at 22 months. Of these ten total transcripts, one appeared in more than one contrast, and none of them was repeated at both times. The complete list of transcripts can be found in Table 3.

When searching for homologies with previously described non-coding RNAs for cattle using the NONCODE database [44], three of the four transcripts in the 15-month group had already been identified (TCONS_00038113 as NONBTAT030133.1, TCONS_00044746 as NONBTAT029274.1, and TCONS_00057377 as NONBTAT031951.1), and in the 22-month group, of the six transcripts, only two were identified (TCONS_00039302 as NONBTAT031112.1 and TCONS_00052474 as NONBTAT027406.1). All of these identifications had over 80 percent identical matches.

### 3.4. lncRNA with Regulatory Potential

Regulatory impact factors were used to identify lncRNAs that could be modulating the expression of genes related to muscle tissue. Using this approach, 25 (6.3%) of the 394 lncRNA were identified as being potential modulators of the expression of these genes. The comparison between NP and PP showed thirteen lncRNAs, of which eight were exclusive, while the comparison of NP and CP treatments revealed seventeen transcripts, twelve of which were exclusive, and five lncRNAs were shared between the two contrasts (Table 4).

### 3.5. lncRNA Co-Expression Networks

**Figure 3:**
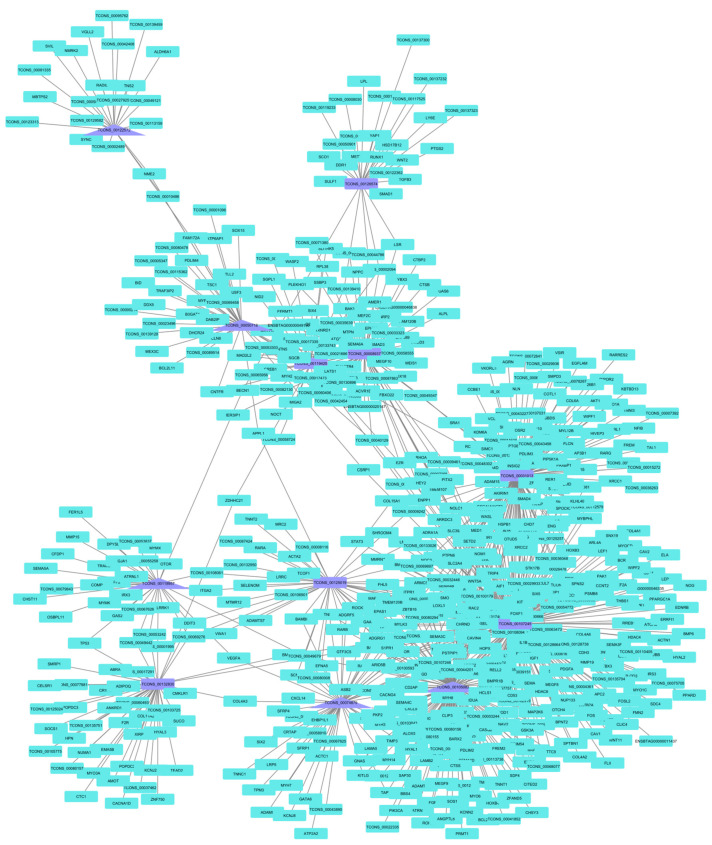
Co-expression network for the PP group based on lncRNA key regulators. The co-expression networks based on the lncRNA key regulators found connections with 478 mRNA in the PP group. These mRNA were identified as being involved in fat-cell differentiation, in utero embryonic development, the transforming growth factor beta (TGF-β) receptor signaling pathway, the semaphorin–plexin signaling pathway, and skeletal muscle tissue development processes.

**Figure 4:**
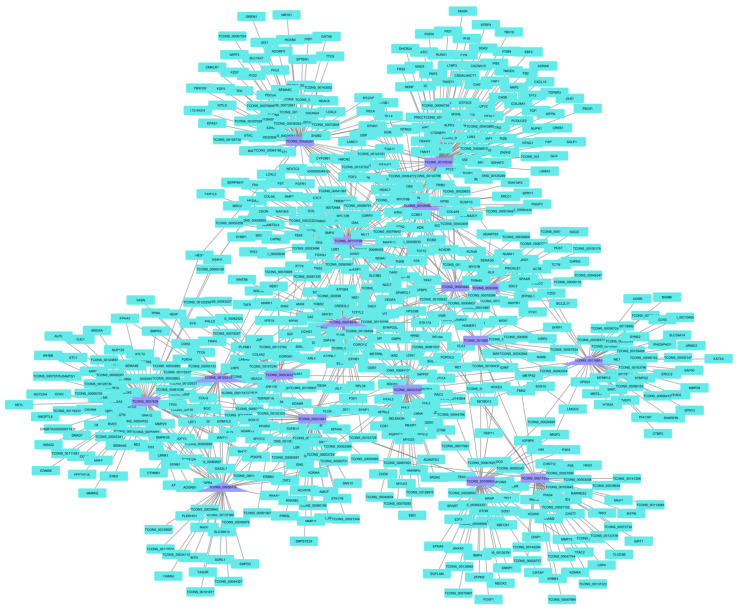
Co-expression network for the CP group based on lncRNA key regulators. The co-expression networks based on the lncRNA key regulators found connections with 495 mRNA in the CP group. After functional enrichment, the involvement of lncRNA in in utero embryonic development, positive regulation of fat-cell differentiation, vasculogenesis, positive regulation of epithelial-to-mesenchymal transition and negative regulation of the canonical Wnt signaling pathway were observed.

When building the co-expression networks for each treatment based on the lncRNA key regulators, the PP group network had lncRNA connections with 478 mRNA (Figure 3). When functional enrichment was performed, the involvement of lncRNA in fat-cell differentiation, in utero embryonic development, the transforming growth factor beta (TGF-β) receptor signaling pathway, the semaphorin–plexin signaling pathway, and skeletal muscle tissue development processes was observed. When looking at the network built by the lncRNA key regulators of the CP group, they connected with 495 mRNA (Figure 4). These mRNA were identified as being involved in in utero embryonic development, positive regulation of fat-cell differentiation, vasculogenesis, positive regulation of epithelial-to-mesenchymal transition, and negative regulation of the canonical Wnt signaling pathway.

## 4. Discussion

In this study, we analyzed transcriptomic data from 15 Nellore young bulls that were subjected to fetal programming to explore the epigenetic mechanisms influencing muscle development throughout both the rearing and termination phases. The findings of this research suggest that there was action of epigenetic regulators of the lncRNA type.

The results found in the literature with regard to muscle development are varied. While some studies suggest the existence of differences in the muscle development of animals [45,46,47], others, which demonstrate similarities between groups [48,49,50], were similar to our findings. This is also repeated for the other characteristics, such as weight and performance. Some studies have already reported similarities between treatments for these traits in steer calves whose mothers were given prepartum supplementation [51,52,53,54,55,56,57].

Although we found no phenotypic differences that indicated changes in muscle development caused by fetal programming [58], it has already been shown that, even without phenotypic differences, there may be changes in gene expression [22]. This occurs because, for there to be phenotypic differences, a priori, a change in gene expression is necessary. Thus, the primary mechanisms through which fetal programming probably begins to show its effects are the epigenetic modifications [11,59]. There is growing evidence that nutritional conditions can alter genome activity through epigenetic modifications [12,60], and several studies using fetal programming have already demonstrated its effects on various organs [61,62], including skeletal muscle [63,64,65,66].

Among the epigenetic mechanisms, three of the main ones are histone modifications, DNA methylation, and gene regulation caused by non-coding RNA [67]. In this sense, there are studies showing the relationship between changes in histone and maternal nutrition [68,69,70], and also their effect on methylation [71,72,73]. Although we did not find genes related to epigenetic mechanisms being differentially expressed, it is possible that their action did not occur in an exacerbated way, and to the point of being detected by RNA-Seq data, although another work has already used this technique [74].

Another way to search for epigenetic changes would be through lncRNA. The search for this category of ncRNA using data obtained by RNA-Seq is already thoroughly discussed in the literature [37,75,76]; a series of filters are applied in order to identify them. However, it is worth mentioning that part of the lncRNA transcript is lost when the RNA-Seq library is assembled using poly-A tail selection [77]. It is possible to say that our search for new lncRNA using RNA-Seq was successful, since when passing through CPC2, less than 20 transcripts (approximately 0.03% of the initial amount) were excluded.

With the new lncRNAs identified, it was possible to perform the differential expression analysis. At the FDR level, only one transcript was found to be differentially expressed. TCONS_00092235 was identified in the contrast between NP and CP treatments for the 22-month analysis. This lncRNA is a transcript located on chromosome 6 in an intergenic region (class code “u”), it is found on the + strand, and it has three exons. TCONS_00030990 is an intergenic region transcript on chromosome 16, which has two exons and is on the—strand. TCONS_00073566 also has two exons, and it is in the intergenic region of chromosome 29. TCONS_00007180 is located in the intergenic region of chromosome 10, and it is on the—strand and has 2 exons. Finally, TCONS_00030818 has two exons, and is in a region that overlaps the IGFN1 gene on the—strand of chromosome 16. The lncRNAs that have already been identified by NONCODE (Table 3) were found in a study that searched for new lncRNA in bovine skin transcriptome [78].

We tried to predict the function of lncRNA key regulators through co-expression networks. In the PP treatment network, function in the TGF-β receptor pathway was identified. This family of proteins is related to the induction of signals that regulate growth, regeneration, differentiation, transformation, and cell death in skeletal muscle [79]. Another identified pathway was the semaphorin–plexin signaling pathway, a network with which 11 semaphorin genes were related. Semaphorin-plexins are related to synaptic signaling, and indirectly related to muscle excitation [80]. Regarding the in utero embryonic development pathway, Ma et al. [81] considered this pathway significantly enriched when comparing lncRNA differentially expressed in muscle samples collected at different stages of animal development. On the other hand, the enrichment of the fat-cell differentiation and skeletal muscle tissue development pathways was expected, since, when performing the analysis, there was a pre-selection of genes related to this tissue.

As for the network of the CP group, the vasculogenesis pathway was enriched. Vasculogenesis occurs when new blood vessels are formed [82], which may indicate a greater need for vascularization in skeletal muscle in the animals in this treatment. Regarding negative regulation of the canonical Wnt signaling pathway, it is an important pathway in skeletal muscle, both in the fetal stage and in adults. Canonical Wnt is associated in adulthood with the differentiation of muscle stem cells [83], and the negative regulation of this pathway may indicate the absence of a need for recruitment of these cells.

In this work, several methodologies were tested, and although we did not find great effects associated with maternal supplementation, we know that these differences can be subtle. Even though these differences are not very expressive in terms of generating differential gene expression, changes may occur in the relationship of genes to each other, depending on the treatment. Given this, we developed the co-expression networks, in order to try to determine some differences between the treatments that were not noticed in any other analysis.

## 5. Conclusions

In an intense search for the epigenetic modifications that could be regulating muscle development in cattle, the treatments were found to be similar when a search was made for epigenetic mechanisms that acted directly on histone modifications and chromatin methylation. Despite this, interesting results were found that suggest that protein–energy supplementation in the prenatal period can influence muscle development through regulation by lncRNA.

## Figures and Tables

**Table 1:** Composition and nutrients of the supplements offered throughout the gestational period of the cows.

Ingredients/Nutrients	Mineral Supplement	Protein–Energy Supplement
Corn (%)	35.00	60.00
Soybean meal (%)	-	30.00
Dicalcium phosphate (%)	10.00	-
Urea 45% (%)	-	2.50
Salt (%)	30.00	5.00
Minerthal 160 MD (%) *	25.00	2.50
Total digestible nutrients (%)	26.76	67.55
Crude protein (%)	2.79	24.78
Non-protein nitrogen (%)	-	7.03
Acid detergent fiber (%)	1.25	4.76
Neutral detergent fiber (%)	4.29	11.24
Fat (%)	1.26	2.61
Calcium (g/kg)	74.11	6.20
Phosphorus (g/kg)	59.38	7.24

* Mineral premix composition (Minerthal company, Sao Paulo, Brazil): Calcium = 8.6 g/kg; Cobalt = 6.4 mg/kg; Copper = 108 mg/kg; Sulfur = 2.4 g/kg; Fluorine = 64 mg/kg; Phosphorus = 6.4 g/kg; Iodine = 5.4 mg/kg; Manganese = 108 mg/kg; Selenium = 3.2 mg/kg; Zinc = 324 mg/kg; Sodium monensin = 160 mg/kg [23].

**Table 2:** Filtering between the treatments applied to generation of the connections.

Filtering	Connections Performed
PP − NP	Connections that appeared only in the PP and not in the NP
PP	Relations exclusive to the PP
CP − NP	Relations from the CP that did not appear in the NP
CP	Relations exclusive to the CP
PP + CP − NP	Relations that appeared only in the PP and CP, and not in the NP

**Table 3:** Differentially expressed long non-coding RNAs.

Period	Contrast	Transcript	Identification	*p*-Value	Adj. *p*-Value
15 m	NP vs. PP	TCONS_00030990		0.0048	0.99
	CP vs. PP	TCONS_00038113	NONBTAT030133.1	0.0017	0.99
		TCONS_00044746	NONBTAT029274.1	0.0052	0.99
		TCONS_00057377	NONBTAT031951.1	0.0077	0.99
22 m	NP vs. CP	TCONS_00092235		2.26 × 10^−7^	0.0001
	NP vs. PP	TCONS_00092235		0.0004	0.19
	NP vs. CP	TCONS_00052474	NONBTAT027406.1	0.0030	0.80
		TCONS_00073566		0.0044	0.80
		TCONS_00007180		0.0062	0.83
	CP vs. PP	TCONS_00030818		0.0085	0.99
		TCONS_00039302	NONBTAT031112.1	0.0037	0.99

**Table 4:** A list of lncRNAs associated with possible key regulation of muscle development, and the number of associated connections on the co-expression network.

Treatment	lncRNA	Identification	Connections
PP	TCONS_00107245	NONBTAT031978.1	247
	TCONS_00105083	-	167
	TCONS_00031013	NONBTAT028263.1	131
	TCONS_00008937	-	74
	TCONS_00074879	NONBTAT028969.1	55
	TCONS_00132830	NONBTAT031353.1	44
	TCONS_00050716	NONBTAT028732.1	42
	TCONS_00125019	-	41
	TCONS_00119425	NONBTAT026662.2	39
	TCONS_00118957	NONBTAT021767.2	34
	TCONS_00126574	NONBTAT031687.1	28
	TCONS_00122572	-	24
	TCONS_00132533	NONBTAT031349.1	-
CP	TCONS_00105330	NONBTAT030235.1	108
	TCONS_00113158	NONBTAT030355.1	87
	TCONS_00078394	-	85
	TCONS_00028261	NONBTAT026662.2	71
	TCONS_00074879	NONBTAT028969.1	68
	TCONS_00118957	NONBTAT021767.2	50
	TCONS_00106901	NONBTAT019405.2	48
	TCONS_00022335	-	47
	TCONS_00031681	-	46
	TCONS_00105083	-	45
	TCONS_00122572	-	43
	TCONS_00017335	-	42
	TCONS_00053837	-	42
	TCONS_00050716	NONBTAT028732.1	42
	TCONS_00063942	NONBTAT028058.1	35
	TCONS_00050901	NONBTAT028721.1	24
	TCONS_00108094	NONBTAT027378.1	24

## Data Availability

The datasets generated during and/or analyzed during the current study are available from the corresponding author on reasonable request.

## Supplementary Materials

The following supporting information can be downloaded at: https://www.mdpi.com/article/10.3390/ani14040652/s1, Table S1: Genes related to epigenetic mechanisms.
