# Supplementary material for: Evaluation of Muscle Long Non-Coding RNA Profile during Rearing and Finishing Phase of Bulls Subjected to Different Prenatal Nutritional Strategies
Source: Animals (Basel). 2024 Feb 18;14(4):652. doi: 10.3390/ani14040652 (PMC10886332; doi:10.3390/ani14040652)
Supplement: Supplementary file 1 [file animals-14-00652-s001.zip › Supplement Files/Table S1.pdf]

Table S1. Genes related to epigenetic mechanisms

DNA Methyltransferases		
DNMT1	DNMT3A	DNMT3B

Histone Acetyltransferases								
ATF2	CDYL	CIITA	ESCO1	ESCO2	HAT1	KAT14	KAT2A	KAT2B
KAT5	KAT6A	KAT6B	KAT7	KAT8	NCOA1	NCOA3	NCOA6	

Histone Methyltransferases							
CARM1	DOT1L	EHMT2	KMT2A	KMT2C	PRMT1	PRMT2	PRMT3
PRMT5	PRMT6	PRMT7	PRMT8	SETDB2	SMYD3	SUV39H1	

SET Domain Proteins (Histone Methyltransferase Activity)								
ASH1L	KMT2C	KMT2E	KMT5A	KMT5B	NSD1	NSD2	SETD1A	SETD1B
SETD2	SETD3	SETD4	SETD5	SETD6	SETD7	SETDB1	SUV39H1	

Histone Phosphorylation						
AURKA	AURKB	AURKC	NEK6	PAK1	RPS6KA3	RPS6KA5

Histone Ubiquitination								
DZIP3	MYSM1	RNF2	RNF20	UBE2A	UBE2B	USP16	USP21	USP22

DNA & Histone Demethylases						
KDM1A	KDM4A	KDM4C	KDM5B	KDM5C	KDM6B	MBD2

Histone Deacetylases					
HDAC1	HDAC10	HDAC11	HDAC2	HDAC3	HDAC4
HDAC5	HDAC6	HDAC7	HDAC8	HDAC9	

SWI / SNF Complex Components				
ARID1A	INO80	PBRM1	SMARCA2	SMARCA4

Polycomb Group Genes								
ASXL1	BMI1	CTBP1	CTBP2	EED	EZH2	PCGF1	PCGF2	PCGF3
PCGF5	PCGF6	PHC1	PHC2	RING1	RNF2	SUZ12	TRIM27	

Chromobox / Heterochromatin Protein 1 (HP1) Homologs						
CBX1	CBX3	CBX4	CBX5	CBX6	CBX7	CBX8

Bromodomain Proteins								
BAZ1A	BAZ1B	BAZ2A	BAZ2B	BPTF	BRD1	BRD2	BRD3	BRD4
BRD7	BRD8	BRDT	BRPF1	BRPF3	BRWD1	BRWD3	WDR11	ZMYND8

Chromodomain / Helicase / DNA-Binding Domain (CHD) Proteins					
CDYL	CDYL2	CHD1	CHD2	CHD3	CHD4
CHD5	CHD6	CHD7	CHD8	CHD9	

Nucleosome-Remodeling & Histone Deacetylase (NuRD) Complex Components					
CHD3	MBD3	MTA1	MTA2	NAB2	SPEN

Plant Homeodomain (PHD) Proteins				
NSD1	PHF1	PHF13	PHF2	PHF21A
PHF21B	PHF3	PHF5A	PHF6	PHF7

Inhibitor of Growth (ING) Family Members					
ING1	ING2	ING3	ING4	ING5	RING1

Methyl-CpG DNA Binding Domain (MDB) Proteins					
HINFP	MBD1	MBD2	MBD3	MBD4	MECP2

CCCTC-Binding Factor (Zinc Finger Protein)
CTCF
